# Functional MRI Study of Working Memory Impairment in Patients with Symptomatic Carotid Artery Disease

**DOI:** 10.1155/2014/327270

**Published:** 2014-02-20

**Authors:** Shasha Zheng, Miao Zhang, Xiaoyi Wang, Qingfeng Ma, Hua Shu, Jie Lu, Kuncheng Li

**Affiliations:** ^1^Department of Radiology, Xuanwu Hospital of Capital Medical University, Beijing 100053, China; ^2^Beijing Key Laboratory of Magnetic Resonance Imaging and Brain Informatics, Beijing 100053, China; ^3^Department of Neurology, Xuanwu Hospital of Capital Medical University, Beijing 100053, China; ^4^State Key Laboratory for Cognitive Neuroscience and Learning, Beijing Normal University, Beijing 100875, China

## Abstract

The neuropsychological tests in patients with internal carotid artery (ICA) demonstrated cognitive deficits associated with frontal lobe dysfunction, but the pathophysiological mechanism of memory impairment is not fully understood. This study evaluated relationship between degree of ICA stenosis and frontal activations induced by working memory (WM) task using fMRI. The fMRI data of 21 patients with unilateral ICA stenosis (left/right, 11/10) and 21 controls were analyzed. In comparison with controls, ICA patients demonstrated significant activations in middle frontal gyrus (MFG) bilaterally, particularly in left MFG. In right ICA stenosis, there was slightly less MFG activation than that of controls. Importantly, lower MFG activity was associated with higher stenosis of ipsilateral ICA. For left ICA stenosis, weaker activation in left MFG was negatively correlated with degree of stenosis. Similarly, for right ICA stenosis, there was a significant negative correlation between right ICA stenosis and weaker activation of right MFG. Cognitive impairments in ICA stenosis were associated with frontal lobe dysfunctions. Left ICA stenosis had worse WM impairments than right ICA stenosis, which was affected by the degree of stenosis.

## 1. Introduction

Cerebrovascular diseases are associated with cognitive decline and dementia. Patients with occlusive diseases of the internal carotid artery (ICA) are at risk for cognitive impairment [1–3]. A systematic review of cognitive disorders in ICA patients finds subtle cognitive deficits in 70% of the studies reviewed [4]. Therefore, some patients may be in a preclinical stage of vascular dementia. Particularly, patients with carotid artery disease who have suffered a transient ischemic attack (TIA) can have lasting cognitive impairment, even without visible ischemic lesions on MRI [5, 6]. Similarly, neuropsychological tests show cognitive deficits in working memory (WM), attention, reasoning, psychomotor speed, and executive functions; frontal lobe dysfunction has been a consistent finding [4–7]. WM is the brain system that maintains a limited amount of information for short periods of time and manipulates that information [8]. The frontal cortex is involved in WM tasks with asymmetric activations in the left and right hemispheres during verbal and nonverbal WM tasks [9, 10]. Furthermore, several studies reported worse memory impairment in patients with left carotid artery disease than those with right carotid artery disease. The different patterns observed argue against that high-grade stenosis of ICA is simply a marker for vascular disease and its risk factors [6, 11, 12]. To date, the cognitive functions of carotid occlusive disease have been assessed using neuropsychological tests [4–7, 11, 13]. Various cognitive functions have been linked to specific brain regions. However, previous neuropsychological tests are not able to precisely reveal cognitive deficits in specific brain regions involving particular tasks. Therefore, the pathophysiological mechanism of memory impairment is not fully understood. Recently, functional magnetic resonance imaging (fMRI) has increasingly been used to study cognitive function in humans. It has been explored for elucidating cognitive impairment mechanisms, especially WM impairment.

We hypothesized that brain dysfunction WM impairment in patients with symptomatic ICA disease; and specifically that the differences in brain dysfunction between left and right ICA disease were associated with the degree of stenosis in ipsilateral ICA, and the functional differences between left and right frontal lobes. Therefore, the purpose of the present study was as follows: (1) to investigate the abnormal frontal activations of digit WM in patients with ICA stenosis/occlusion and ipsilateral TIA and (2) to investigate the relationship between the activations in the frontal and the degree of ICA stenosis using fMRI.

## 2. Subjects and Methods

### 2.1. Subjects

This study was comprised of 49 consecutive patients who were assessed for neurocognitive effects of symptomatic carotid artery disease in the Department of Neurology. Symptomatic carotid stenosis is defined as stenosis having caused ischaemic events in the ipsilateral eye (transient monocular blindness) or cerebral hemisphere (transient ischaemic attack or stroke) in the past 6 months [14, 15]. The study protocol was approved by the local Institutional Review Board and written informed consent was obtained from all of subjects. 21 patients with symptoms of transient ischemia (lasting <24 hours) were enrolled. As determined by the use of a transcranial Doppler (TCD) or angiography, these patients demonstrated high-grade stenosis (70–99%) or unilateral internal carotid artery (ICA) occlusion. All patients had experienced at least one TIA and symptoms had occurred, at most, 6 months before inclusion in the study. All patients had normal intelligence; however, their total memory scale scores (*M* = 72.54 ± 21.96) on the Clinical Memory Scale (CMS) were lower than the healthy subjects (*M* = 88.54 ± 14.57) [16, 17]. The handedness of the subjects was assessed using the Edinburgh inventory [18]. The exclusion criteria included left-handedness; contralateral ICA occlusion or high-grade stenosis (≥70%); large infarct infarction or multiple lacunar infarctions (≥3) on MRI; severe white matter lesions (≥ Grade 3) on MRI, especially lacunar infarcts involved middle frontal gyrus and white matter lesions overstep the immediate subependymal region of the ventricles; history of other brain diseases; deafness and/or blindness. The severity of leukoaraiosis (LA) was graded using the visual rating scale proposed by Sakakibara et al. [19]. These patients were divided into two groups: left ICA stenosis or occlusion (*n* = 11, age range from 39 to 75 years, mean age 59.45 ± 11.72 years) and right ICA stenosis or occlusion (*n* = 10, age range from 38 to 70 years, mean age 56.10 ± 10.86 years) based on the TCD or digital subtraction angiography (DSA) study. The control group consisted of 21 healthy volunteers (age range was 33–69 years and mean age was 54.64 ± 11.85 years). These subjects were age- and education-matched to the patients (age: *t* = 1.67, *P* > 0.05; education: *t* = 1.74, *P* > 0.05). In the control group, carotid artery disease and intracerebral lesions were excluded by TCD and MRI examinations. All subjects were administered a battery of neuropsychological tests involving auditory digital memory and visual digital memory [20, 21]. All patients were found to have impaired WM compared with the control group. The demographic data of the study subjects were shown in Table 1.

### 2.2. MRI Data Acquisition

Scanning was performed on a 3.0-Tesla whole-body scanner (Trio Tim, Siemens). A T2-weighted (TR/TE: 3830 ms/98 ms; flip angle: 180°; slice thickness 5 mm; gap: 5 mm; FOV: 230 mm × 218 mm; matrix: 179 × 320) image was acquired for exclusion of intracranial lesions. High-resolution 3D magnetization prepared rapid gradient echo imaging (MPRAGE) and anatomical images (TR/TE: 1970 ms/3.93 ms; flip angle: 15°; thickness 1.70 mm; gap: 0.85 mm; FOV: 250 mm × 250 mm; matrix: 448 × 512) of the entire brain were obtained before the functional images were acquired. A T2*-weighted gradient-echo echo-planar imaging (EPI) sequence was used to acquire functional images with 30 axial slices (TR/TE: 2000/30 ms; flip angle: 90°, thickness: 5 mm; gap: 0 mm; FOV: 240 × 240 mm; matrix: 64 × 64).

### 2.3. Working Memory Tasks

**Figure 1:**
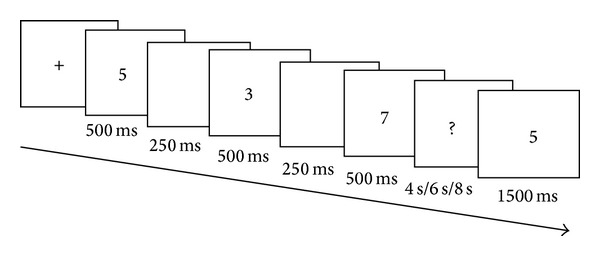
Digit working memory task.

All subjects were required to perform a 3-item delayed-match-to-sample task with digit items [22, 23]. A fast event-related design was adopted. For the digital task the stimuli, ten different one-digit numbers (0–9), were projected randomly onto the center of a screen on the head coil. Subjects responded by pressing a keypad with their thumbs as quickly and accurately as possible. Prior to the MRI examination, all subjects were trained to perform 2 practice trials to ensure that they fully understood the tasks. A single trial of this digit working memory task is schematized in Figure 1. At the beginning, subjects were required to memorize a set of three items sequentially. Each item was shown for 500 milliseconds (ms) on the center of the screen with two 250 ms blank intervals in between each item. After the third item disappeared from the screen, an interruption mark was presented for 4 s, 6 s, or 8 s, respectively. Subjects were told to focus on the screen and hold the stimulus items in mind. The duration of the interruption mark presentation was randomly selected. Finally, a probe appeared for 1500 ms that contained half of the items presented in the previous set. Participants were instructed to press a button to decide whether or not the probe was the same as one of the three previously presented items (left hand indicated “Yes” and right indicated “No”). Reaction time and accuracy of the response were recorded during the scans for each subject. There were intertrial intervals (ITI) that consisted of a presentation of a blank screen. These intervals were used as a baseline epoch. The duration of ITIs ranged from 2 s to 14 s. The duration of each task was 8 min and 28 s and included 30 trials.

### 2.4. fMRI Data Analysis

The fMRI data were preprocessed and statistically analyzed using the Analysis of Functional Neuroimages (AFNI) software [24]. The first four scans for each participant were excluded from data to minimize the transit effects of hemodynamic responses. Functional images were corrected for head motions by aligning all volumes to the fifth volume using a six-parameter rigid-body transformation. Statistical maps were spatially smoothed with a 6 mm full width at half maximum (FWHM) Gaussian kernel.

We analyzed the brain activities of working memory relative to the baseline of the resting period during the ITI. Individual anatomical images and functional t-maps were coregistered to the standard Talairach and Tournoux space [25]. All images were resized to 3 mm × 3 mm × 3 mm voxel. At the group level, the threshold of group maps was set at a voxel level of *t* > 2.040 (*P* < 0.005, number of voxels > 14) with a spatial extent correction. This threshold corresponded to an overall *α* < 0.05 of family-wise error rate, as calculated with AlphaSim (http://afni.nimh.nih.gov) for all intracranial voxels in the image volumes. Previous WM studies have found that the bilateral frontal lobes play an important part in processing WM information [9, 10, 23, 26, 27]. For this reason we focused on the frontal lobes as our regions-of-interests (ROIs), to explore the relationship between the behavioral data of WM and the functional activation of ICA disease. The ROIs were defined functionally as spheres with a 6 mm radius on the basis of activation clusters in the bilateral frontal lobes from the group analysis (see Section 3). The peak activation coordinates from the cluster of the contrast analysis were selected as the center of each ROI. Then, these ROIs were employed as masks to extract the mean percent signal change (averaged over the ROI) in the blood oxygen level dependent (BOLD) response.

### 2.5. Statistical Analysis

Neuropsychological data were analyzed using SPSS 11.0 computer software (SPSS Inc., Chicago, IL). The characteristics of patients and controls were compared using analysis of variance (ANOVA). The correlations between response time (RT), response accuracy (RA) of digit WM task, activation intensity within the defined frontal ROI, and the degree of ICA stenosis were analyzed using a Pearson's correlation.

## 3. Results

All subjects completed the fMRI digit task. The left and right ICA stenosis or occlusion patients showed significantly weaker (left: *P* = 0.032; right: *P* = 0.041) and less accurate responses (left: *P* = 0.039; right: *P* = 0.043) than those of the control subjects (Table 2). Higher degrees of left ICA stenosis were positively correlated with RT (*r* = 0.412, *P* = 0.042), but not with RA (*r* = −0.243, *P* = 0.436). Higher degrees of right ICA stenosis were not correlated with either RT (*r* = 0.247, *P* = 0.424) or RA (*r* = −0.108, *P* = 0.671).

**Figure 2:**
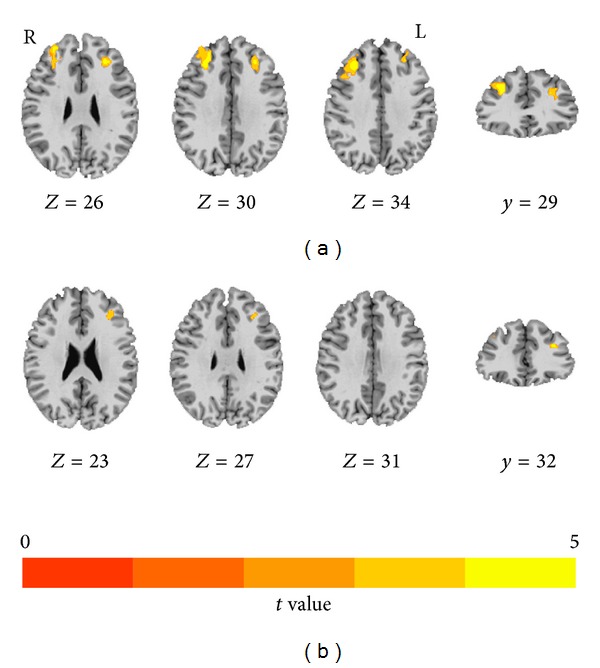
fMRI results for a digit WM task. (a) The left ICA patients showed significantly less activation in the left MFG as well as slightly less activation in the right MFG than control subjects. (b) The right ICA patients showed slightly less activation in the right MFG only than control subjects.

The control group revealed a domain area of activation in the bilateral middle frontal gyrus (MFG), frontal gyrus, and supplementary motor area involving digit WM task. The peak of the activation was located in the bilateral MFG. The digit WM task induced significantly asymmetrical activations in the MFG (peak coordinate: (−31, 31, 23), *t* = 5.915) and volume (475 voxels) in the left MFG. The left and right ICA groups showed similar activity clusters compared with those of the control group. MFG activations for digit WM task in subjects were listed in Table 3. A direct comparison between left ICA patients and control subjects revealed that the patients had significantly less activations in the left MFG and slightly less activations in the right MFG (Figure 2(a)). Right ICA patients demonstrated slightly less activation in the right MFG than control subjects (Figure 2(b)).

**Figure 3:**
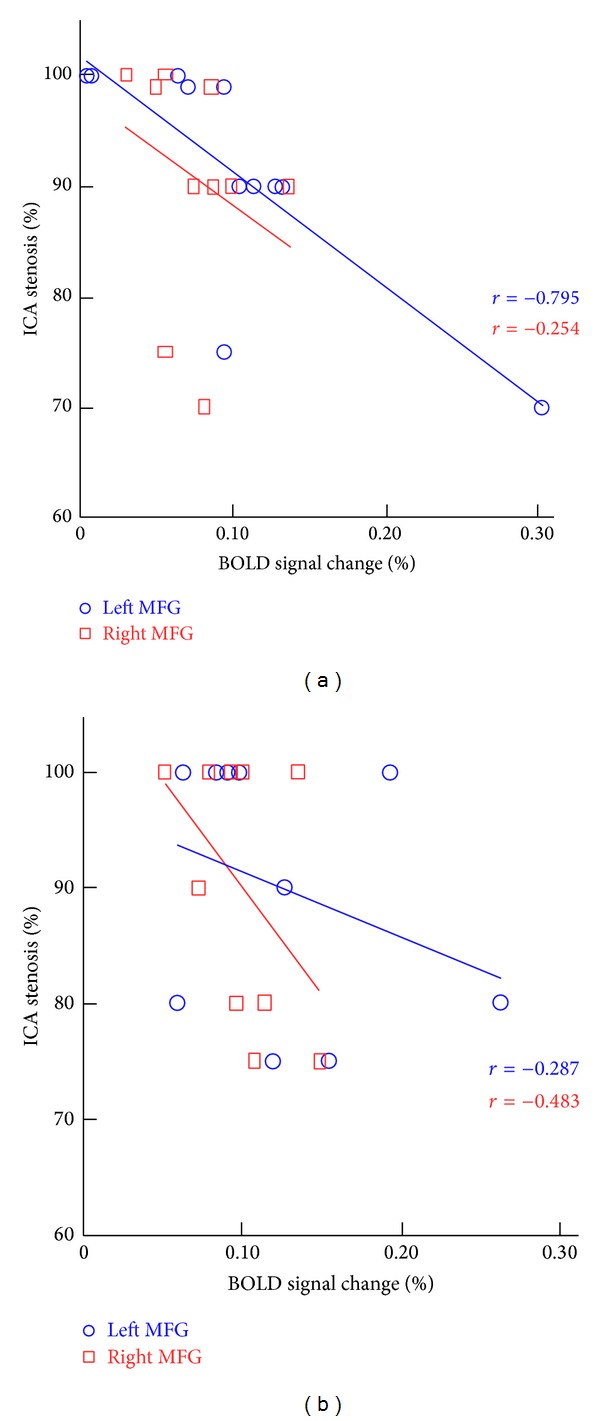
Relationship between MFG activation and the degree of ICA stenosis. (a) A significantly negative correlation was found between left ICA stenosis and lower activation intensity in the left MFG (blue), but not in the right MFG (red). (b) A significantly negative correlation was found between right ICA stenosis and lower activation intensity in the right MFG (red), but not in the left MFG (blue).

We selected the bilateral MFG for further ROI analysis. For the left ICA patients, there was a significant negative correlation between ICA stenosis and activation intensity in the left MFG (*r* = −0.795, *P* = 0.009), but not in the right MFG (*r* = −0.254, *P* = 0.264). By contrast, there was a significant negative correlation between the right ICA stenosis and activation intensity in the right MFG (*r* = −0.483, *P* = 0.041), but not in the left MFG (*r* = −0.287, *P* = 0.218). Correlation graphs of MFG activation and ICA stenosis are displayed in Figure 3.

## 4. Discussion

The results from this study suggested that patients with left ICA disease have more severe frontal lobe dysfunctions than those of age- and sex-matched controls. Compared with controls, the right ICA patients found slightly less activation in the right MFG. Such weaker MFG activity was also associated with a higher stenosis of ipsilateral ICA. A significant negative correlation was found between left ICA stenosis and activation of left MFG in the left ICA patients. Similarly, there was a significant negative correlation between the right ICA stenosis and activation of right MFG in right ICA patients.

Behavioral studies have reported impaired frontal lobe function in patients with ICA disease. Patients demonstrated WM impairments by neuropsychological assessments [4–7, 11, 13]. In addition, results from fMRI and positron emission tomography (PET) imaging suggest that the frontal cortex plays a critical role in the WM [9, 10, 23, 26, 27]. MFG is the core region involving WM. Our results indicated that the activation of MFG (especially left MFG) is key region participant in digit WM. However, compared with controls, MFG activation was weaker in patients with left or right ICA disease. This finding demonstrates the ability of fMRI to detect abnormal frontal lobe activation in patients with mild cognitive impairment.

ICA disease may cause cognitive impairment but the mechanisms involved are poorly understood. Our results suggest that frontal lobe dysfunction may be one of the possible mechanisms. Since fMRI is based on hemodynamic coupling in activated brains, our results also imply that perfusion responses may be involved. Previous studies propose that compromised frontal lobe perfusion may be a cause of cognitive impairment in patients with ICA disease. Thus there is the suggestion that restoring, or at least improving, frontal perfusion with carotid endarterectomy or carotid artery stenting may enhance cognitive function [28, 29]. In this study, we did not examine changes in cerebral blood flow in our subjects. Future studies combining fMRI with perfusion imaging may be helpful for investigating this hypothesis.

Two forms of WM, namely, verbal and nonverbal WM, have been found to be asymmetrically represented in the left and right frontal cortex; however, this left-right specialization is relative [9, 10]. The digit WM is one of the most frequently used verbal WM tasks. Prior WM studies of digit task ability revealed activation within the MFG [23, 26]. In this study, digit WM in the control group demonstrated increased fMRI activation in the left MFG. This pattern of asymmetric activation in the MFG was disrupted in patients with left ICA disease. These patients presented with decreased activations in the bilateral MFG, especially in the left MFG, compared with the control group. In contrast, patients with right ICA disease retained the asymmetric pronounced left activation in the MFG. fMRI in the patient group showed no significant difference in the left MFG compared with the control group; however, there was less activation in the right MFG of patients. These fMRI results suggest that the left side of the ICA may reflect the left dominant frontal cortex in digit WM. For verbal WM, frontal dysfunction was worse in patients with left ICA disease than those with right ICA disease. Our results are consistent with previous neuropsychological findings which have reported that a higher degree of stenosis of the left ICA was associated with cognitive deficits and cognitive decline in the left cerebral hemisphere. However no such correlation was observed in right ICA stenosis [6, 11]. Additionally, even asymptomatic patients with left ICA stenosis appear mainly to have verbal deficits [11, 30].

A graded relationship has been shown between some neuropsychological tests and the degree of stenosis [31]. In the present study, the degree of left ICA stenosis was positively correlated with RT of digit WM. No correlation was found in right ICA disease. It is noted that the speed of decision making was reduced in patients with left ICA disease. The degree of left ICA stenosis was associated with lower activation in the left MFG, whereas the degree of right ICA stenosis was associated with lower activation in the right MFG. Previous fMRI studies of letter WM have suggested that increased intimal-medial thickening of the carotid wall is associated with lower signal intensity in MFG [27]. Viewed in combination, these findings not only suggest ICA stenosis as an independent risk factor for cognitive impairment, but may also be consistent with the idea that the degree of ICA stenosis may be a marker of cognitive decline in symptomatic patients.

In conclusion, our study suggests that cognitive impairments may be related with frontal dysfunctions in patients with symptomatic ICA disease. In the present study, patients with left ICA disease demonstrated worse verbal WM impairments due to more severe left frontal dysfunction. We also found that the degree of ICA stenosis may affect the severity of WM impairment. Further studies are warranted, perhaps utilizing multimodality MRI techniques such as perfusion and spectroscopy, in order to elucidate the mechanisms and markers of cognitive impairment in patients with symptomatic ICA disease.

## Figures and Tables

**Table 1:** Demographic characteristics of patients and controls.

	Left ICA (*n* = 11)	Right ICA (*n* = 10)	Control (*n* = 21)
Age, years (mean ± SD)	59.45 ± 11.72	56.10 ± 10.86	54.64 ± 11.85
Gender M/F	8/3	3/7	11/10
Education, years (mean ± SD)	10.36 ± 4.03	11.60 ± 2.72	12.00 ± 2.95
Auditory digital memory (mean ± SD)	88.48 ± 8.16	89.63 ± 7.39	97.34 ± 3.28
Visual digital memory (mean ± SD)	90.37 ± 6.02	91.44 ± 6.71	98.02 ± 3.15
Severity of vessel stenosis			
70–99%, *n*	8	5	/
Occlusion, *n*	3	5	/
Hypertension, *n*	5	4	/
Hypercholesterolemia, *n*	1	2	/
Diabetes, *n*	1	1	/
Heart disease, *n*	1	1	/
Smokers, *n*	2	2	/
White matter lesions, *n*	6	5	/

**Table 2:** Reaction time and accuracy of a digit working memory task in all subjects.

	Left ICA (*n* = 11)	Right ICA (*n* = 10)	Control (*n* = 21)
RT (ms)	1159.33 ± 310.28*	1099.83 ± 208.19*	983.28 ± 107.34
RA (%)	82.61 ± 19.42*	83.21 ± 20.67*	97.14 ± 4.32

Data are presented as mean ± SD. *P < 0.05 compared with controls.

**Table 3:** Medial frontal gyrus activations for a digit working memory task in subjects.

	Left MFG			Right MFG		
	Voxels	Peak (*x*, *y*, *z*)	*t* value	Voxels	Peak (*x*, *y*, *z*)	*t* value
Control	475	−31, 31, 23	5.915	294	34, 46, 32	5.085
LICA	241	−22, 46, 29	4.791	47	22, 19, 35	3.221
RICA	152	−34, −1, 47	3.506	15	−28, 1, 53	3.036
Control-LICA	129	−28, 34, 23	3.341	217	28, 28, −32	3.915
Control-RICA	26	−31, 31, 23	2.962	21	34, 46, −29	2.521

MFG: medial frontal gyrus; LICA: left internal carotid artery; RICA: right internal carotid artery.
